# Molecular Profiles of Brain Metastases: A Focus on Heterogeneity

**DOI:** 10.3390/cancers13112645

**Published:** 2021-05-28

**Authors:** Shan Ali, Zuzanna Górska, Renata Duchnowska, Jacek Jassem

**Affiliations:** 1 Department of Oncology and Radiotherapy, Medical University of Gdańsk, 14 Smoluchowskiego St., 80-214 Gdansk, Poland; shanali@gumed.edu.pl; 2 Department of Oncology, Military Institute of Medicine, 128 Szaserów St., 04-141 Warsaw, Poland; zgorska1@student.umb.edu.pl (Z.G.); rduchnowska@wim.mil.pl (R.D.)

**Keywords:** brain metastases, cancer, genomics, heterogeneity, precision medicine, targeted therapy, immunotherapy, lung cancer, breast cancer, melanoma

## Abstract

**Simple Summary:**

Precision cancer medicine depends on the characterization of tumor samples, usually by a single-tumor biopsy, to administer an optimal therapeutic. However, primary tumors and their metastases are often heterogeneous. A metastatic lesion may harbor a completely different genetic makeup to that of its parent tumor, and a single tumor sampling may be ineffective in selecting the most efficient therapy. Brain metastases, due to their low availability and specific microenvironment, pose a particular challenge for precision medicine. In this review, we highlight the genetic landscape of brain metastases, with a particular focus on their heterogeneity. To illustrate this problem, we present phenotypic alterations in brain metastases originating from lung cancer, breast cancer, and melanoma. This article may help clinicians better understand alterations in brain metastases and the relevance of their heterogeneity.

**Abstract:**

Brain metastasis is a common and devastating clinical entity. Intratumor heterogeneity in brain metastases poses a crucial challenge to precision medicine. However, advances in next-generation sequencing, new insight into the pathophysiology of driver mutations, and the creation of novel tumor models have allowed us to gain better insight into the genetic landscapes of brain metastases, their temporal evolution, and their response to various treatments. A plethora of genomic studies have identified the heterogeneous clonal landscape of tumors and, at the same time, introduced potential targets for precision medicine. As an example, we present phenotypic alterations in brain metastases originating from three malignancies with the highest brain metastasis frequency: lung cancer, breast cancer, and melanoma. We discuss the barriers to precision medicine, tumor heterogeneity, the significance of blood-based biomarkers in tracking clonal evolution, the phylogenetic relationship between primary and metastatic tumors, blood–brain barrier heterogeneity, and limitations to ongoing research.

## 1. Introduction

The brain houses intricate processing centers that are defended by a unique set of neuroprotective safeguards and microenvironments. Despite the brain’s natural defenses, brain metastases (BMs) are relatively common and accompany about 6% of all cancers [1]. Brain metastases are 10 times more common than primary brain tumors [2]. The most common primary sites for BMs are lung cancer (40–50%), breast cancer (15–25%), and melanoma (5–20%) [3,4]. 

Systemic treatment options for BMs are limited. Due to insufficient blood–brain barrier (BBB) penetration, the efficacy of traditional cytotoxic agents in BMs is generally lower than that in extracranial sites. Molecularly targeted therapies have been shown to provide better control of both intracranial and extracranial disease. However, patients with BMs have been frequently excluded from clinical trials investigating these agents. 

Today’s personalized cancer medicine relies on identifying a tumor’s molecular characteristics via a tumor biopsy sample; this will help doctors to better target the cancer treatment. However, the heterogeneity of individual and metastatic tumors poses an obstacle. Several sequencing analyses have shown inter- and intratumor heterogeneity (i.e., spatial diversity between several tumors and spatial or temporal diversity within a single tumor, respectively), which may lead to treatment failure [5,6,7,8]. Thus, the usefulness of a single tumor sample in characterizing a tumor’s mutational landscape may be limited. Indeed, metastatic sites may exhibit unique genomic alterations and epigenetic differentiation and may be influenced by their local microenvironment [9]. 

Currently, decisions regarding systemic therapies for patients with BMs are still mainly based on the biomarkers assessed in the primary tumor. Consequently, targeted therapies for BMs remain suboptimal and under-utilized. This review will discuss the barriers to precision cancer medicine related to BM heterogeneity, limitations of BM research, and clinical implications of BM molecular profiles. To illustrate this problem, we will specifically discuss the molecular profiles of BMs originating from three malignancies with the highest BM frequency: lung cancer, breast cancer, and melanoma. Our article should be considered a narrative review, as no profound analysis of the literature on this topic was attempted.

## 2. Functional Heterogeneity and the Darwinian Model

Tumor heterogeneity may result from natural or therapeutic selection (based on the Darwinian model and on branched evolutionary tumor growth) [10,11,12]. In the Darwinian model, therapeutic drugs or the tumor microenvironment (e.g., hypoxia, growth factors) act as selective pressure by eliminating cellular clones with specific genomic and epigenetic alterations or microenvironmental features (in a specific stromal niche). This process leads to the survival of the fittest clones and contributes to clinical resistance to chemotherapy and targeted drugs [10,11,12]. Branched tumor evolution (also known as phylogenetic evolution) induces intratumor heterogeneity, making targeted approaches problematic, as the tumor cells that survive are the same that keep proliferating. For example, Brastianos et al. (2015) [13] showed that, although the BMs and primary tumors shared the same progenitor, they still diverged autonomously. In this study, 53% of BMs harbored potential clinically actionable mutations not detected in the matched primary tumor samples [13]. However, spatially and temporally separated BMs were genetically homogenous.

## 3. Challenges in Clinical Research on Brain Metastases

The tissue sampling of BMs poses a particular challenge, as many patients are not candidates for brain resections or have tumors in inaccessible sites. The low availability of tissue samples makes designing comprehensive studies problematic. These limitations may also lead to the underestimation of current statistics on BMs [14]. Moreover, there is a potential selection bias, as larger tumors may be diagnosed more often than smaller tumors due to their symptoms, caused by mass effect and the compression of nearby structures. Thus, smaller tumors may be underreported and understudied. 

Magnetic resonance (MR) imaging remains the modality of choice to evaluate patients with BMs. However, MR images of primary tumors and infections may mimic BMs and smaller tumors may be missed. Additionally, the increasing size of BMs may not necessarily indicate tumor recurrence but may be, for example, due to pseudoprogression after radiotherapy. Thus, better prognostic and predictive imaging markers are needed to identify and monitor tumors. More specialized imaging techniques, such as diffusion tensor imaging, perfusion-weighted imaging, and MR spectroscopy can effectively differentiate primary from secondary tumors [15]. Positron emission tomography has shown promise in differentiating radiation necrosis from tumor recurrence [15]. 

The brain’s distinct microenvironment results from its complex functional anatomy, the population density of local cell types (i.e., neurons versus glia), vascularization, lymphatic architecture, and oxygen requirements. Therefore, a metastatic tumor in one brain location may have a drastically different microenvironment to its neighbor, only millimeters away. Such spatial heterogeneity poses a challenge when objectively comparing BMs [16]. 

BMs harbor a diverse set of cell-intrinsic mechanisms that may significantly alter their growth. Moreover, BMs may have confounding effects from prior therapies, creating further variability. Lastly, most studies use archival formalin-fixed material, primary tumor datasets with brain relapse data, or experimental models. Experiments based on in vivo samples would be more valuable and would provide a more accurate depiction of BMs’ pathophysiology. 

Another problem hindering the clinical research on BMs is that such patients are often excluded from clinical trials [17]. However, in 2019 the US Food and Drug Administration published guidelines that encouraged the inclusion of patients with BMs in future trials (Docket ID FDA-2019-D-0357). One more limitation hampering BM research is economical. Most cancer research and public attention worldwide is geared towards primary cancers, with only about 5% allocated for metastases, even though, in some cases, metastases are far more common and are the leading cause of cancer mortality [18]. Over the past three decades, primary cancer survival rates have improved, whereas outcomes for metastatic cancer have remained stagnant [19]. It is apparent that more funding and greater social awareness should be dedicated to cancer metastasis research. 

## 4. Genomic Profiling of Brain Metastases Using Circulating Tumor DNA 

The targeted gene therapy of BMs relies on the genetic profiling of a primary tumor and its corresponding BMs. Due to spatial and temporal heterogeneity, biopsies are often repeated to identify the full spectrum of genetic alterations of a cancer. In the case of BMs, obtaining several biopsies poses an even greater challenge, since the procedure is invasive and carries the risk of complications. In some cases, the tumor’s location may be inaccessible and the patient may be in poor physical condition and thereby unable to tolerate the procedure. Alternatives to direct BM biopsy, including sampling the primary tumor, regional lymph nodes, or extracranial metastases, have proven to be unreliable [13].

Irrespective of the source of traditional tissue biopsy, it analyzes only a small part of a much larger malignant tumor. This limitation may be overcome by analyzing cell-free circulating tumor DNA (ctDNA), which represents the entire tumor mass. Plasma ctDNA analysis has shown promise in characterizing genomic alterations of BMs and monitoring tumor response to therapy in several studies [20,21,22]. Moreover, liquid biopsy is non-invasive, less traumatic, feasible regardless of the anatomic location, and straightforward to collect and analyze; this makes initial tumor analysis and future monitoring relatively fast. Studies have shown that mutations not found in traditional tissue biopsies may be detected by liquid biopsies [23,24], which supports their role in clinical practice. 

Another source of genomic material in patients with BMs is cerebrospinal fluid (CSF). Mattos-Arruda et al. (2015) [21] showed that ctDNA derived from CSF is more abundant than that from plasma; thus, CSF ctDNA may be preferred to genomically characterize BMs and monitor treatment response. Pentsova et al. (2016) [25] used CSF as a source of ctDNA in 32 patients with BMs from solid tumors and in 12 patients with primary brain tumors. They detected and monitored somatic alterations in oncogenic kinases that allowed the tumors to progress despite kinase inhibitor therapy. These data support the use of ctDNA to obtain a comprehensive molecular profile to target BMs, monitor treatment, and surveil for genomic alterations.

Whereas studies using CSF paint a promising outlook for ctDNA, most have used small cohorts and are retrospective [26]. Thus, the exact role of this approach in clinical practice is ill-defined and it is unclear whether ctDNA analysis may lead to improved patient outcomes. Moreover, most CSF DNA is derived from healthy non-malignant cells and tumor DNA constitutes an extremely small fraction of the total; this necessitates modifications in sequencing assays to achieve an adequate sensitivity for analysis [25]. Using a lumbar puncture to acquire CSF ctDNA may not always be feasible, especially in space-occupying BMs that increase the risk of herniation. In some situations, suboccipital puncture (also known as cisternal puncture) may be an alternative to lumbar puncture. The analysis and interpretation of ctDNA are variable, since there is no one established protocol for sampling, handling, and analysis. Thus, using different protocols and detection technologies on the same patient may yield different results [27]. Further, comparing results from various laboratories may be problematic, since experimental details are only partially reported. Several ongoing prospective studies may help to shed light on the implementation of CSF ctDNA as a surrogate for a BM biopsy sample and provide molecular profiles of brain metastases in major malignancies [26].

## 5. Brain Metastases Heterogeneity in Major Malignancies

### 5.1. Lung Cancer

Brain metastases occur at diagnosis in 10% to 20% of patients with lung cancer, and another 40% to 50% of patients will develop BMs during their illness [28]. The occurrence of BMs is highest in small-cell carcinoma (80%), followed by squamous cell carcinoma (20%), adenocarcinoma (18%), and large-cell carcinoma (11%) [29]. Due to their high prevalence, adenocarcinomas account for over half of all BMs from NSCLC [14,29]. 

Few studies have evaluated the genetic features of BMs in lung cancer. In the study of Villalva et al. (2013) [30], molecular alterations of *KRAS, EGFR,* and *ALK* were present in 39% (30/77), 3.9% (3/77), and 7.7% (1/13) of BMs, respectively. In the study of Nicoś et al. (2018) [31], among 10 analyzed genes at least one abnormality was found in 59 cases (41%), including *KRAS* in 21%; *EGFR* in 6.2%; *ALK* in 4.8%; *DDR2* in 2.1%; *PIK3CA* in 2.1%; *NRAS* in 1.4%; and *HER2*, *AKT1*, *PTEN*, and *MEK1* in 0.7% of patients each. The reported concordance rate of the *EGFR* mutations in paired primary NSCLCs and BMs ranges between 36% and 100% (Table 1) [32,33,34,35,36,37,38]. Hence, testing *EGFR* mutations in the primary site of NSCLC may not be informative for planning the use of *EGFR* tyrosine kinase inhibitors (TKIs) for the treatment of BMs. A 2011 review of eight studies found that the first-generation EGFR TKIs, gefitinib and erlotinib, induced a high response rate in BMs from *EGFR*-mutated NSCLC [37]. The efficacy of third-generation EGFR TKI osimertinib seems to be higher compared to that of first-generation TKIs [39,40,41]. EGFR TKIs may also decrease the risk of BMs in lung cancers harboring *EGFR* mutations [38,40,42].

About 3% of BMs from NSCLC harbor ALK translocations and 11% harbor ALK amplifications [43]. The ALK gene most commonly fuses with the EML4 gene [44]. The first-generation ALK inhibitor crizotinib and the second-generation inhibitors alectinib, ceritinib, bigatinib, and ensartinib are effective in treating NSCLC with activating ALK translocations; however, their efficacy in BMs shows significant differences [45,46,47]. Among the currently identified secondary ALK mutations L1196M, G1269A, S1206Y, F1145C, and S1206Y, some are associated with resistance to crizotinib and sensitivity to ceritinib, ensartinib, and alectinib [48]. Other mutations (C1156Y and F1174L) are sensitive to alectinib and ensartinib, but not to ceritinib. In turn, the I1171T mutation is sensitive to ceritinib and ensartinib, but not to alectinib. Finally, the G1202R mutation has a confirmed sensitivity to a new third-generation ALK inhibitor, lorlatinib. In the ALEX study, the CNS-specific objective response rates for alectinib and crizotinib in the first-line treatment following previous radiotherapy were 36% and 29%, respectively [49]. For patients without previous radiotherapy, the CNS response rates for these compounds were 74% and 24%, respectively [49]. The time to CNS progression was also significantly longer with alectinib than with crizotinib (HR 0.16; *p* < 0.001). Further, lorlatinib has shown a high activity in treating and preventing BM (response in BM after crizotinib, alectinib, and bigatinib was 87%, 53%, and 54%, respectively) [50]. In the CROWN trial comparing first-line lorlatinib and crizotnib, the cumulative incidence of CNS progression was 3% and 33%, respectively [51]. Such a spectacular efficacy of lorlatinib may be attributed to eliminating preexisting subclones harboring ALK resistance mutations or preventing their emergence.

Paik et al. (2016) [52] conducted the next-generation sequencing (NGS) of primary sites and matched BMs in 79 squamous cell lung carcinomas. They found that truncal *PTEN* loss in primary tumors increased the risk of BMs. BMs demonstrated extensive genetic heterogeneity and clonal differences with their primary sites. Ma et al. (2018) [53], in a study of 28 lung adenocarcinomas and matched BMs, found 43 variants of seven genes: *TP53*, *EGFR*, *CTNNB1*, *PIK3CA, SMAD4, KRAS,* and *B-RAF*. The same potentially actionable mutations in BMs were not present in the primary tumor, indicating that there was a high degree of genetic heterogeneity [53]. Wang et al. (2019) [54] performed a retrospective NGS in 61 primary NSCLCs and matched BMs. In this series, over 80% of the cases showed a high concordance for most common drivers (*EGFR, KRAS, TP53*, and *ALK*) between the primary tumors and the corresponding BMs, whereas the genes encoding the *CDK4/CCND1, CDKN2A/2B,* and *PI3K* signaling pathways were enriched in BMs. Additionally, patients with activated *PI3K* signaling in primary tumors had a significantly shorter BM-free survival. Thus, the identified genomic alterations in BMs could serve as prognostic markers and therapeutic targets. 

Using WES and targeted panel sequencing, Liao et al. (2018) [55] found a high genetic heterogeneity between primary NSCLCs and the corresponding BMs; however, the mutations in the oncogene EGFR and the tumor suppressor gene TP53 remained clonal. This suggests that these genes may be instrumental in BM formation and that most mutations detected in the primary tumor or metastases are sufficient for clinical decisions. In another WES study, BMs exhibited higher somatic variants and chromosomal mutation burden than primary NSCLCs, particularly in genes associated with lung cancer (e.g., KRAS, ROS1, and STK11) [56]. The small study of Li et al. (2020) [57], also using WES, found a high mutation consistency between primary lung adenocarcionoma and BM, but large differences between individuals. The mutation of FAM129C and ADAMTSs and high amplification of NKX2-1 were correlated with the risk of BM [57]. Additionally, the copy number deletions of SAMD2 and SAMD4, associated with the TGF-beta signaling pathway in both primary tumors and BMs, seem to be a therapeutic target in BMs from lung cancer [57].

Liu et al. (2020) [58] performed a single-cell RNA sequencing data analysis of 50 primary lung adenocarcinomas and their corresponding BMs. They discovered a significant intratumoral heterogeneity in both the BMs and parent cancers. The pathways related to translational initiation, endoplasmic reticulum stress, exosomes, and unfolded protein response were upregulated in BMs compared to the primary sites [58]. Despite the higher mutation burden in BMs than in the primary NSCLCs, the latter were reported to harbor a greater richness of T-cell clones than their paired metastases [59]. The vast majority of T-cell clones were specific to a single lesion, with minimal overlap in T-cell clones between paired lesions. These differences highlight the challenge of immunotherapy in NSCLC patients with BMs.

### 5.2. Breast Cancer

The frequency of BMs with multiple extracranial metastases in particular subtypes of breast cancer is 31% for triple-negative, 28% for HER2, 20% for luminal A, and 13% for luminal B [60]. The estrogen receptor α (ERα), progesterone receptor (PR), and epidermal growth factor receptor 2 (HER2) statuses determined in the primary tumor are often not maintained in their metastatic sites.

The extent of receptor conversion between primary breast cancer and matched BMs varies considerably [61,62,63,64,65]. In a study including 120 primary tumors and their matched BMs, ERα, PR, and HER2 conversion in BMs occurred in 29%, 29%, and 14% of cases, respectively [66]. Another study using genome-wide DNA methylation profiling demonstrated distinct epigenetic signatures in BMs, allowing their categorization according to the primary tumor and providing therapeutically relevant information [67].

In experimental murine HER2-positive breast cancer, significant differences in BM appearance were found by magnetic resonance imaging, histology, and immunohistochemistry across the three experimental models [68]. De Mattos-Aruda et al. (2018) [69], using the high-depth targeted sequencing of 254 genes frequently mutated in HER2-positive breast cancer and/or related to DNA repair, demonstrated significant spatial and temporal genetic heterogeneity between primary breast tumors and their BMs. The mutations in cancer genes *FGFR2*, *PIK3CA,* and *ATR*; homozygous deletion in *CDKN2A*; and amplification in *KRAS* were restricted to BMs. Another analysis showed a higher concordance of the HER-2 status between the primary tumor and its BMs using fluorescent in situ hybridization (FISH) compared to using immunohistochemistry [70]. This information may empower pathologists to test the HER-2 status of the primary tumor with FISH if obtaining a sample from the BM is impossible. The heterogeneity of BMs in breast cancer may also include BBB permeability. Adkins et al. (2016) [71] showed distinct differences in passive BBB permeability between five experimental murine models of BMs. These changes were distinct even within the same metastatic lesion from the identical cell line. Another mouse model of breast cancer showed that distinct clones within the same tumor may carry heterogeneous metastatic behavior [72]. The tumor colonies that mimic endothelial/vascular cells show a high metastatic potential [72] and particular brain tropism [73]. The analysis of the BM vascular permeability may provide additional information on metastatic heterogeneity and allow targeting therapy (e.g., with anti-vascular agents), tracking treatment response, and monitoring disease progression [72,73,74,75,76,77]. 

Moreover, the heterogeneity of the BBB may impair drug transport and delivery across the vessel wall to reach its target cancer, thwarting targeted therapies and other systemic therapies. Several molecular, biological, and physical methods have been proposed to improve transport across the BBB [78]. For example, a phase II study found that ANG1005 (a lipoprotein receptor-related protein 1-targeting peptide bound to paclitaxel) increased delivery and median survival in breast cancer patients with BMs [79].

Blood-based biomarkers such as circulating tumor cells (CTCs) provide an attractive non-invasive alternative to tissue biopsies and may allow the evaluation of the current genomic features of a tumor. A study including 57 breast cancer patients analyzed DNA from individual CTCs, and corresponding primary tumors and BMs [80]. CTCs were detected in one third of the cases, of which 60% were *EGFR*- and keratin-positive, and 40% were only keratin-positive. The presence of CTCs carried an adverse prognosis. A comparative NGS analysis performed in three patients showed the similar features of CTCs and matched primary tumors, but also identified alterations in pathways known to be important in the formation of BMs. The high clonality of CTCs in one patient indicated a strict clonal selection of cells competent for BMs. 

Ramani et al. (2019) [81] extracted CTCs from seven patient-derived orthotopic xenograft models of triple-negative breast cancer. Shed CTCs were present in 86% (32/37) of the models. Individual cells present within the same cluster exhibited heterogenous cytokeratin (an epithelial marker), vimentin (a mesenchymal marker), and mixed cytokeratin/vimentin phenotypes. Thus, CTC analysis may serve as a useful tool for investigating breast cancer heterogeneity.

BMs create a neuroinflammatory response with reactive microglia and astrocytes [82,83]. In breast cancer, DNA double-strand break repair genes *BARD1* and *RAD51* are overexpressed in BMs compared to the parent tumors [84]. Their activation may be a protective reflex to the reactive oxygen species-mediated genotoxic stress caused by BMs. The overexpression of *BARD1* and *RAD51* speaks to the heterogeneity of BMs caused by their microenvironments.

### 5.3. Melanoma

Metastatic melanoma is managed with a combination of immunotherapy (CTLA-4 checkpoint inhibitor ipilimumab and PD-1 inhibitors nivolumab and pembrolizumab), targeted therapy (BRAF inhibitors vemurafenib, dabrafenib, and encorafenib), and chemotherapy (dacarbazine and temozolomide). The MAPK-ERK pathway, which includes *BRAF*, *NRAS*, *MEK1/2,* and *ERK1/2*, is instrumental to the evolution of melanoma [85].

Fischer et al. (2019) [86], using RNA sequencing, showed a higher immunosuppression and enhanced oxidative phosphorylation gene expression in BMs compared to extracranial melanoma metastases. IACS-010759, an oxidative phosphorylation inhibitor, is currently a subject of early-phase clinical trials in melanoma BMs. 

Heitzer et al. (2019) [87] established a melanoma BM cell line MUG-Mel1 and two resulting clones, D5 and C8, which harbored slight differences. Using several analytical techniques and experimental in vitro and in vivo models, they showed unique differences between particular lines in terms of their morphology, lipidome, growth behavior, surface, and stem cell markers.

Colombino et al. (2012) [88] demonstrated that brain and skin melanoma metastases presented a lower consistency of *BRAF*/*NRAS* mutation status than lymph node and visceral metastases, suggesting the evolution of independent subclones in select cases. Simonsen et al. (2015) [89] exposed the intertumor heterogeneity in the vascularity and invasiveness of artificial melanoma BMs in mice. These differences were related to the varying expression of the angiogenic factors, vascular endothelial growth factor A, interleukin 8, and the matrix metalloproteinases 2 and 9. Compared to liver and lung metastases, melanoma BMs are characterized by a higher AKT activation and lower PTEN expression [90]. Hence, a site-specific activation of signaling pathways should be considered when developing new targeted treatments. 

Using whole-exome and RNA sequencing in a patient with metastatic acral melanoma, Lee et al. (2020) [91] showed that the brain microenvironment, rather than immune escape mechanisms within a tumor, drives the resistance to immune and targeted therapies. These data indicate the important role of site-specific microenvironments in facilitating resistance and the potential value of salvage treatments considering a tumor’s interactions with its microenvironment. 

Izraely et al. (2020) [92] explored the role of aldolase C (a glycolytic enzyme normally expressed by astrocytes and neurons) in shaping the malignant phenotype of melanoma cells. They found that aldolase C may both induce or inhibit the malignant phenotype in particular brain-metastasizing variants. This study confirms the importance of the brain microenvironment and the highly varying tumor responses related to intertumor heterogeneity.

## 6. Conclusions

Tumor heterogeneity poses several challenges for cancer management. In many instances, single-tumor biopsy sampling may not be representative of metastatic lesions that have undergone different mutation processes. A more comprehensive assessment of tumor portraits considering both inter- and intratumor heterogeneity may inform individual treatment decisions and increase opportunities for personalized medicine. Information about BMs’ molecular features offers the potential to screen for new innovative therapies. However, the routine assessment and quantification of BM heterogeneity remain a logistic and clinical challenge. Novel techniques, such as the analysis of blood-derived markers, may allow for significant progress to be made in this field.

## Figures and Tables

**Table 1:** *EGFR* mutations in paired primary tumors and brain metastases.

Author	*EGFR* Activating Mutations in BMs	Concordance in Matched Pairs
Gow [32]	39% (18/67)	36% (4/11)
Matsumoto [33]	63% (12/19)	75% (6/8)
Wojas-Krawczyk [34]	6.3% (9/143)	100% (32/32)
Munfus-McCray [35]	40% (4/10)	High concordance
Han [36]	60% (3/5)	80% (4/5)

Abbreviations: EGFR—epidermal growth factor receptor; BMs—brain metastases.
